# On Some Scientific Problems of Dental Surgery

**Published:** 1883-04

**Authors:** C. S. Tomes


### THE DENTAL REGISTER.

VOL. XXXVII.] APRIL, 1883. [No. 4.

### COMMUNICATIONS.

### On some Scientific Problems of Dental Surgery.

BY MR. C. S. TOMES, F.R.S.*

It is a reproach of long standing that the method and practice of medicine are unscientific, and that they are merely empirical, whilst the practitioners of medicine in the early days of the separation between medicine proper and surgery in their turn looked down upon the surgeons as even less scientific than themselves. It was not long before the surgeons made such strides in their art that they could claim full equality with the physician, as rendering useful service to their patients; but, more particularly where handicraft forms an important element in treatment, there long lingered a tendency to draw a distinction between the so-called "practical" and the "scientific" man, to the detriment of the latter. Other and far abler pens than mine have demonstrated the fallacy which underlies such distinctions by pointing out that "scientific" knowledge differs from any other knowledge in no respect but in that a rigorous accuracy of fact is demanded, and vague beliefs incapable of proofs are rejected; so that to say a word against science in our practice is simply to plead the advantages of ignorance. The immense triumphs which have been achieved by modern surgery, triumphs which are being added to every day, are the outcome of the pursuit of more rigorously scientific methods, and if we in our specialty of dental surgery would advance likewise, we can only hope to do so by following a similar course.

* Read at the Annual General Meeting of the Association at Liverpool, August 26th, 1882.

So much I have said by way of prelude, in order to remove any impression that might exist that "scientific problems" lead to no practical result. I am sorry to say that a review of recent dental literature brings sadly into prominence the lack of true scientific spirit amongst our workers; it is voluminous enough, it is positive enough, but the voluminousness and the positiveness alike are the outcome of insufficient observation, insufficient experiment, and insufficient exactitude of thought. Take for example the discussion as to those physical qualities of gold which we term cohesiveness and non-cohesiveness, or take the much discussed questions arising out of amalgams and their defects; and of all that has been written there is not one hundreth part which is worth reading, or which can possibly carry conviction to any one's mind. It is always, "I am sure," "I have all my life known," or something to that effect, and very rarely an experiment, or statistics, or anything convincing. And when we have statistics or experiments, they are too often such as to demonstrate by internal evidence that little reliance can be placed upon them. In this depressing state of things it has seemed worth while, although having little in the way of research to communicate, to formulate exactly what we want and need to know about some of the conditions we are most often called upon to treat, namely, about alveolar abscess and its causes.

Surgeons, and physicians too, are at the present time largely engaged in investigating what is termed the "germ theory of disease," and this has, as you all know, a most practical bearing upon treatment. That aspect of the question which mainly concerns us may be broadly put thus; whether certain morbid conditions can and do arise within the body itself, or whether they are imported from without, in the form of germs or what not. It would be entirely out of place to here attempt to give any outline of the basis upon which antiseptic surgery rests, but it may be well to remind my hearers of some few of those experiments and observations which bear most directly upon the problem before us.

It is thoroughly well established that organic fluids or tissues, though eminently susceptible of decomposition, do not undergo putrefactive decomposition without the help of organisms, bacteria and micrococci, which grow in them, and act apparently as ferments. If these are excluded, as they are from an aseptic flask, or are killed by boiling, as is the case of tinned meat and vegetables, no putrefaction takes place. According to Dr. Ogston micrococci do not produce putrefaction, and flourish best when removed from the air; on the other hand bacteria cause stinking putrefactions.

Portions of tissues cut from animals just killed, and passed, with full antiseptic precautions, into an aseptic flask, may be kept fresh for an unlimited time; and this leads to the inference that the germs of micrococci and bacteria do not circulate in healthy blood, and do not exist in healthy organs. But introduce a trace of dust into the flask, and the whole contents at once putrefy, whilst the putrid fluid is found to swarm with organisms. So also the discharges from a wound kept aseptic do not putrefy, and the patients do not get pyemia or septicæmia, except through the failure of the antiseptic precautions. Another most instructive experiment is being performed every day on a large scale. In the south of Europe the castration of male animals is affected by an operation termed "bistournage;" roughly speaking this consists of twisting the testis upon the spermatic cord, so that without any exposure to external influences at all it is killed, and instead of putrefying it withers away. This portion of tissue, killed by the severance of its vessels is analogous to the pulp of a tooth which has been killed by a blow, to which I shall revert. But Professor Chaveau has carried this experiment farther; he has introduced bacteria into the veins of animals upon whom bistournage was about to be performed, with the result of bringing about the decomposition of the killed testis, which then became the cause of profuse suppuration; so that the proof is in this case exceedingly complete that a portion of tissue may be devitalised without decomposing and without setting up inflammation around it to any material extent so long as it is not exposed to external influences; but once introduce septic organisms into it, even by way of the circulation, and this happy result is no longer attainable.

One might multiply examples of this fact to any extent, but I

will merely remind you that surgeons, in subcutaneous operations, have for years availed themselves of the immunity from subsequent ill-results afforded by the avoidance of any exposure to outside contamination. In septicæmia, micrococci may generally be detected in the blood, as they also may in several morbid contions which do not concern us now.

It some time ago occurred to me that we had, in the numerous cases of abscess which come before us, the opportunity of studying, to considerable advantage, the share which organisms take in these productions, but I have not since been able to make my observations sufficiently numerous to draw any general principles from them. So far as I know there is no literature on the subject of the connection of organisms with dental abscess, with the exception of allusions to alveolar abscess in a report by Dr. Ogston (*British Medical Journal*, March, 1881). He found micrococci and bacteria in the pus from aveolar abscesses; and generally speaking, he found organisms in the pus of all acute abscesses which he examined, but did not find them in that of "cold" abscesses. We are not told in his paper of what nature the alveolar abscesses examined were, whether acute or chronic. Amongst the many important results in Dr. Ogston's report, which will repay the most careful study, is this: that injection into animals of pus which contained no micrococci produced no result, but that of pus containing micrococci, or of micrococci artificially cultivated, always produced abscess, though if thoroughly carbolized it did not do so.

The pulp chamber of a tooth is a cavity which, prior to the occurrence of caries, is absolutely isolated from external influences, and has but one channel into it, namely, its apical foramen: even after the occurrence of caries it can be tolerably easily and completely closed. And hence it is a particularly favorable place for investigations of this nature, and, it might be hoped, would lead to results of wide application.

It has been shown by Messrs. A. Underwood and Milles that bacteria are to be found within the substance of carious dentine, and this fact remaines established, whatever view may ultimately be adopted as to the extent of their influence upon the morbid process. The observation that bacteria are not to be found within healthy dentine, which, therefore, even in the absence of enamel, constitutes an efficient barrier, and that they are to be found in carious dentine, is one that has an important bearing upon the present inquiry.

For our present purpose we may consider alveolar abscess as occuring under three sets of conditions:—1. Where the pulp of a tooth has been somewhat nearly approached by caries, a filling has been inserted, and, after the lapse of an uncertain time, the tooth pulp is found to be dead and decomposed. 2. Where the pulp has been killed by a blow, with no breach in the hard tissues of the tooth. 3. Where after removal of the debris of the pulp, and filling or dressing of the roots, abscess recurs after the lapse of considerable time, and without failure of the filling.

I have placed first death of the pulp as a sequence to caries and filling of the tooth, because the problem there involved is a simple one, and also because I have more positive evidence to offer upon this set of conditions, than upon the others.

I have purposely excluded all observations upon cases in which an alveolar abscess had already broken, because in these there was a communication with the exterior of the gum, through which the germs might have entered. Of cases in which I was able to open up the pulp chamber, prior to the occurrence of abscess, I have only met with six since my attention has been directed to the subject, and in every one of these six I found the offensive fluid debris to contain bacteria and micrococci. But it is not always easy to be certain whether micrococci are or are not present, as there is always a great deal of granular matter which renders their discrimination less certain than that of the rod-shaped forms. As I have before mentioned it is not difficult to account for their presence, seeing that carious dentine affords a home for them, and that there may have even been a minute actual exposure of the pulp. But it is a question of great interest, and of practical importance too, to discover, if possible, what share the organisms have had in provoking the inflammation of the pulp which has preceded its death, and in the subsequent changes in the way of its decomposition.

In the admirably clear lecture of Professor Burdon Sanderson on Inflammation (reported in the *British Medical Journal* for April, 1882), it is related as proven that in the cornea, its epithelium being damaged, an inflammation springs up which is due to the entrance of germs and their multiplication in its substance; and it is worth noting that in these exceptional cases in which the early extraction of the tooth has, for some reason or other, been resorted to, and we get the opportunity of examining the pulp prior to its entire death, we always find that the point of inflammation and disintegration corresponds to the point of complete or approximate exposure, and that a stinking decomposition has been set up at this point, while the rest of the pulp is alive, and, as far as we can see, healthy, beyond this zone of inflammation. So far it looks as though something getting in had done it, as though microzymes had entered, and the inflammation were comparable to that in a cornea of which the surface had been damaged; actual observations on the presence of bacteria at this stage are, however, wanting, and they are much needed. I have only a single observation to record, in which I found the bacteria abundant.

On the other hand, as Professor Burdon Sanderson well insists, the view that all inflammations are due to organisms is quite untenable, and inflammation may spring up at once in a part which has been subject to damage, without the possibility of the entrance of germs; so that we are by no means driven to the absolute conclusion that the bacteria which I found were the causes of the inflammation. But these inflammations which are the result of physiological "damage" to a part, elsewhere come to a kindly ending. In his own words "an uncomplicated inflammation is neither reproductive nor inflective, neither benign nor malignant; if it has any tendency it is to leave off as soon as the occasion for it passes." Therefore the tooth pulp, supposing that it really has inflamed before its death, should not on that account give rise to products capable of poisoning the parts about the apical foramen.

At page 293, he says, "I endeavored to show, on the ground of experiment, that the only inflammations to which minute organisms stand in relation are those which, from their proved dependence on previously existing inflammation, may be properly termed secondary or infective; and consequently that the organisms in question were not so much mischief-makers as mischief-spreaders." That is to say, although an inflammation may come into existence without their aid, their presence communicates to it, after it has come into existence, the power of reproducing itself in previously healthy tissues, whether by extension or dissemination. These facts he sums up: "(1) That the exudation of a normal inflammation is not effective; (2) that no organisms endowed with inflammation-producing functions exist in the atmosphere or in ordinary aqueous liquids with which our bodies come in contact; (3) that whenever an inflammation becomes infective, it owes that property to chemical change in the exudative liquid, of which the presence of microzymes is a necessary condition." Now if we attempt to apply these canons to dental diseases, we are at once struck with the fact that the contents of the pulp chamber of a tooth are in a sense virulently infective, for the escape of the smallest quantity from the apical foramen sets up a most violent inflammation and abscess, which however, only very rarely spreads further, (though in one of my cases in which the patient was long in seeking relief there was almost erysipelatous inflammation of the face attended with considerable fever) but there is, so far, no difficulty in understanding how the tooth pulp may have complied with the conditions laid down by Professor Sanderson, as to a prior inflammation, the exudation of which, with the presence of organisms, has become infective. But it is a matter of some moment to us to discover, if possible, whether we have most often to deal with an inflammation caused by the invasion of the pulp by microzymes, or set up by irritation, for in the one case the profuse and persevering use of antiseptics is indicated, in the other sedative applications, and this point can only be elucidated by very numerous and very careful observations.

Passing now to our second group we have by accident most instructive experiments not rarely brought under our notice, when the pulps of front teeth which have been severely struck die, although the hard tissues of the tooth are quite intact. Here we have a portion of tissue dead, which has in no way been exposed to external agencies. No germs can have entered it from without, and it is in this respect in the same position as the testis killed by "bistournage," or as a portion of tissue cut out and introduced into a flask with antiseptic precautions. It therefore might fairly be expected to remain mummified without undergoing any putrefactive decomposition ; and this does sometimes happen, a case of this kind having come under my own notice since my attention has been specially directed to the subject.* But much more frequently, after an interval of exceedingly variable length, violent alveolar abscess ensues, and on opening up the tooth by drilling into it we find the contents of the pulp cavity stinking, whilst the violence of the alveolar abscess betokens the virulence with which the tissues adjacent to the apical foramen have been infected.

It becomes a matter of the greatest interest, to discover whether organisms do or do not play a part in this sequence of events, and the solution of this problem has a bearing on questions far wider than the field of dental surgery. And the importance of the experiment is almost equally great, whether it turns out that bacteria or micrococci are there, or not. For if there, they must have got there by way of the circulation in a healthy individual, and must have been (1) present in the tooth pulp prior to the accident, or (2) have entered it during the brief period of inflammation which we know clinically to sometimes intervene between the injury and the death of the pulp, or else, if the death of the pulp has been instantaneous (3) have crept in by the apical foramen subsequently, a hypothesis which does not commend itself as probable.

On the other hand, if, after extended and numerons observations, no organisms prove to be there, we shall have proof of a putrefactive decomposition with stinking and poisonous products taking place without the intervention of bacteria or micrococci; in other words, that which, according to the doctrines of modern surgery, ought to be in the condition of an aseptic slough, becoming septic.

* And since the reading of this paper, I have met with two central incisors, the pulps of which had been killed four years previously ; there was tenderness and slight fullness over the apices of the roots, and the pulps themselves were coherent, white and sodden, but without any offensive smell. No bacteria were found.

One does not meet with these cases very frequently, and it would require a large number of observations to prove a negative, yet so far I have not succeeded in quite satisfying myself of the presence of either micrococci or bacteria in any case which I have examined; in one case I found a few bacteria, but they were so few, and so unequally distributed over the slide that I suspected their not having been derived from the fluid from the pulp chamber. The question, then, for the present remains open, but I would point out that the occurrence of alveolar abscess after the death of a pulp killed by a blow is very often a slow process, extending over months or years, or never happening at all, whilst an abscess usually follows very speedily on the heels of the death of a pulp which has been nearly or quite exposed.

And whilst it is comparatively easy to find bacteria, or be satisfied of their abscence, it is very different with the round micrococci, for the debris of these pulps is almost made up of granular material.

The third group of cases are those which are most troublesome in our daily practice, those cases, namely, in which alveolar abscess recurs after we have done what we can in the way of treatment. It is, of course, a familiar fact that if we can pump creosote through the apical foramen and out through a fistulous opening on the gum, that alveolar abscess may be readily and speedily cured in almost every case, and this would seem to point to its being kept up by organisms. As a matter of fact I have frequently found them present in the pus, and again sometimes I have failed to find them; but I attach little importance to this, inasmuch as I have found them abundantly one day, and on the next, the abscess having meantime greatly subsided, have found very few.

The cases, however, which are most important, and most troublesome are those in which the roots have been thoroughly treated with antiseptics, and filled, say with cotton wool, saturated with creosote. The tooth has remained quiet for a considerable time, and then becomes tender, and an abscess threatens. On removing the filling the wool from some one of the roots is found discolored and offensive. It is hardly to be supposed that organisms have got in subsequently to the filling by way of the tooth, so here again we are in face of alternatives: either we have that which is generally believed never to occur, a putrefactive decomposition without the intervention of organisms, or the germs have escaped the creosote, lain latent for a time, and then developed.

It is a familiar observation that cold will provoke an inflammation about a heretofore quiescent dead tooth; this would be intelligible enough as an instance of a "damage" to a part already perhaps below its standard of health, but whence come the stinking products which leak into the roots of the tooth if there are no microzymes?

And these products we know to be infective, for if in our manipulation we pump anything, no matter how little, through the apical foramen, we set up an acute abscess. Of these cases I have not had the opportunity of examining a sufficient number to draw any conclusions, but I may remark that the late Dr. Dean, in a paper on alveolar abscess, read before the International Medical Congress, laid much stress on the application of the rubber dam to a tooth in which an alveolar abscess had been cured prior to the removal of the dressing; and he was advocating these "antiseptic precautions," although speaking purely from the clinical point of view. I myself asked him the question, prior to the reading of the paper, whether he advocated this precaution for a theoretical reason, or as a result of practical experience, and he told me purely on account of the latter. He saw, then, from his clinical experience the necessity of excluding something: of its nature he was uncertain.

On the other hand, if we are contending with living organisms, it is strange that we cannot secure more uniform success, for we are able to bring into play antiseptics with a directness and in a strength impossible elsewhere in the body.

To recapitulate briefly it is believed that:—

(i.) Putrefaction with the development of stinking products depends upon the presence of organisms.

(ii.) Inflammation may be directly caused by organisms, or it may arise independently of them.

(iii.) Infective inflammations are associated with the presence of organisms.

And we find that:—

(i.) Tooth pulps nearly exposed by caries inflame, die, putrefy, and infect the parts around the apex of the root, and that organisms are found in the debris of the pulp and in the pus of the subsequent abscess.

On the other hand that:—

(ii.) Tooth pulps never exposed to external contamination die, putrefy, and infect the parts outside them; and that organisms have not, so far, been satisfactorily demonstrated in them.

And again:—

(iii.) The dressings in the roots of dead teeth, under circumstances which would render the multiplication of organisms unlikely, do become saturated with offensive fluid.

But against this last we may set the fact that success in the treatment of alveolar abscess seems to be about proportionate to the completeness with which antiseptics can be brought into contact with all the surfaces concerned.

I feel that I owe this meeting an apology, for bringing before them so imperfect an investigation: but there is one excuse for my doing so, and that is, that it is an investigation which to be at all fruitful of results must be carried out by many observers, and, so far as I know, attention has not been very definitely directed towards it. I hope, for my own part, when my observations have become sufficiently numerous, to publish them in detail, and that, by that time, there will be many others to confirm or disapprove them. It may well be that the pursuit of this line of investigation will lead to most practical results in dental surgery, for if it be the case that rigid antiseptic precautions are our only road to success, we have excellent means of applying them by the use of the rubber dam. I may mention that I have lately always, when practicable, removed devitalized pulps under a pool of eucalyptus oil, so that this may run in as the pulp is removed, and no septic contamination take place during this part of the operation; but as good results are ordinarily attainable without this precaution, it is too soon to speak of its value.

On the other hand, failing a practical result for dental surgery, the investigations cannot fail to be of value in throwing light upon a great question of pathology; for the isolation of the pulp chamber of a tooth eliminates a great many of the sources of error and uncertainty which beset similar research upon other organs of the body.

